# Estimating US Earnings Loss Associated with COVID-19 Based on Human Capital Calculation

**DOI:** 10.3390/ijerph19021015

**Published:** 2022-01-17

**Authors:** Fuhmei Wang, Jung-Der Wang

**Affiliations:** 1 Department of Economics, College of Social Science, National Cheng Kung University, Tainan 701, Taiwan; 2 Department of Public Health, College of Medicine, National Cheng Kung University, Tainan 701, Taiwan; 3 Department of Occupational and Environmental Medicine, College of Medicine, National Cheng Kung University Hospital, National Cheng Kung University, Tainan 704, Taiwan

**Keywords:** employment ratio, survival function, COVID-19, productivity loss

## Abstract

Infection with COVID-19 could result in lockdown, quarantine of contacts, absenteeism from work, and temporary productivity loss. This research aims to calculate (1) how the pandemic affects on-the-job probability and earnings for the working population, and (2) how much productivity loss is associated with self or a family member sick with COVID-19. Based on data collected from the U.S Research and Development Survey (RANDS), this research projects the relationship between on-the-job possibility and age of the index group and calculates the employment possibilities of the index group relative to the healthy group, namely the employment ratio. The weekly loss of productivity, presented by earnings, associated with COVID-19 for groups aged 18–44 years and 45–64 years was calculated, since the 18- to 64-year-old population is an economy’s active workforce. Analytical results indicate that the older the age group, the lower the on-the-job possibility, and the higher the weekly productivity loss due to self or a family member being sick from COVID-19. For the group aged 45–64 years, the employment ratio of the index group relative to the healthy group dropped from 0.863 to 0.39, corresponding to a weekly productivity loss of 136–590 US dollars. The overall impact would be about a 9% loss in GDP. Infected or quarantined people would be confined to working in relatively isolated offices or places to allow for social distancing. Proactive health promotion in the workplace plus reactive work through telecommunication systems would reduce such losses. Such preparedness needs to be implemented early for more vulnerable workers who are of middle or old age and/or those comorbid with diabetes.

## 1. Introduction

The coronavirus disease 2019 (COVID-19) pandemic has resulted in severe health concerns and global economic crises, and the United States has been one of the hardest hit countries. Extensive studies have been conducted investigating the various patterns of clinical symptoms, transmission routes, and control throughout this pandemic [1,2,3,4,5,6,7]. The high mortality rate of COVID-19 infection has resulted in a major impact on life expectancy [8,9,10], concentrated especially among the elderly [11,12], and there have been harmful effects on the productivity of the working population [7]. To better understand the influences of this pandemic on economic performance, it is worthwhile to assess the burden of this disease by investigating loss in productivity.

### 1.1. Literature Review

To assess the socio-economic burden of the COVID-19 pandemic, adopting a broad societal aspect is essential, since it is not only concerned with temporary human capital loss due to absenteeism from work but also the permanent productivity loss due to severe premature mortality rates [13]. In addition, we must include the absence of work that resulted from quarantine or isolation because of close contact with infected patients, such as family members and/or caregivers [14]. Because each country has different societal challenges, studies have investigated the cost of absenteeism [15,16,17] or expected losses of gross domestic product (GDP) [18,19,20,21,22,23]. The former could be regarded as the cost of sick patients who are temporarily disabled or deceased plus those who are healthy but under quarantine/isolation, while the latter could present the economic impact of such. While absence of work directly related to illness may be quantified by estimating the number of patients within the working age range under the unit of disability-adjusted life years [13], comprehensive exploration of the societal financial burdens due to isolation and/or quarantine of close contacts in the workplace or at home seems to be lacking and should be included. This research aims to estimate the differences in on-the-job possibility and productivity of human capital between those infected with or exposed to COVID-19 and the healthy population.

The COVID-19 fatality rate is estimated at around 4% among infected patients, but varies across different ages and between different countries, ranging from 0 to 20% [12,21]. Such a high fatality could result in the reduction of life expectancy and affect an economy’s production [13]. Fortunately, thanks to the extensive work performed by scientists and pharmaceutical companies, the rollout of vaccinations is already underway around the world [22]. As research from a healthcare perspective is starting to reveal significant findings, it is now time to pay attention to the economy and the significant consequences the pandemic has had in this context.

### 1.2. The Purpose

Although microbiology has made tremendous advances during the last century, the human capital impact of the COVID-19 pandemic is huge due to the lack of quick and effective control measures, and deserves investigation for future preparedness.

### 1.3. Objectives

This research aims to calculate (1) how the pandemic affects on-the-job probability and earnings for the working population, and (2) how much productivity loss is associated with oneself or a family member sick with COVID-19. Study results could serve as a guide for quantifying economic and demographic burdens.

### 1.4. The Hypothesis of the Research

This research proposes the hypothesis that the productivity loss associated with COVID-19 based on human capital calculation would increase with working age due to oneself or a family member being infected and therefore unable to work during quarantine.

This research aims to calculate (1) how the pandemic affects on-the-job probability and earnings for the working population, and (2) how much productivity loss is associated with being sick or having a family member sick with COVID-19. Study results could serve as a guide for quantifying economic and demographic burdens. Our study contributes to and improves on earlier work in several ways. First, we projected the relationship between age and the possibility of loss of work when oneself or a family member is sick with COVID-19 (12). Second, this study is among the first to integrate survival function with on-the-job possibility to represent employment status to estimate the differences in the productivities of human capital between the healthy and index groups. No studies have explored the employment ratios of people and their families sick with COVID-19 relative to healthy control groups over the pandemic period. Third, we calculated the weekly loss of productivity or earnings for different groups aged 18 years and over based on US weekly wages during the surveyed dates.

## 2. Materials and Methods

### 2.1. Research Methodology Definition

To estimate human capital loss more comprehensively, this research aimed to include the losses not only from the mortality and morbidity of patients themselves but also those that resulted from the quarantine/isolation of family members or caregivers. Namely, we took an alternative approach by estimating the survival function of the general population in 2018 based on the life table as the reference group, which was adjusted for COVID-19 mortality in 2020 (i.e., before versus during COVID-19). Then, the survival function of 2020 was multiplied with the employment possibility in that year captured by the three CDC funded surveys. The difference in survival functions would reflect the permanent productivity loss due to mortality and the temporary productivity loss due to absenteeism would be estimated from the surveyed results indicating the employment loss under the pandemic.

### 2.2. Calculation of Employment Status

The U.S Research and Development Survey (RANDS) recently conducted a survey about the population’s inability to work due to having COVID-19 or a family member being sick with COVID-19. Three rounds of surveys were conducted over the periods of 9 June 2020–6 July 2020, 3 August 2020–20 August 2020, and 17 May 2021–30 June 2021, including, in order, 6794, 5974, and 5450 individuals aged 18–44 years, 45–64 years, and 65 years and over, respectively. This source of high-quality data could reflect work limitations due to hospitalization or quarantine during COVID-19 [12]. Based on the data collected from RANDS, this research projects the relationship between on-the-job possibility and age of the index group and calculates the employment possibilities of the index group relative to the healthy group, namely the employment ratio. The weekly loss in productivity, presented by earnings, is associated with COVID-19 for groups aged 18–44 years and 45–64 years was calculated, since the 18- to 64-year-old population is an economy’s active workforce. Table 1 presents the sample size, the percentages of loss-of-work due to self or a family member being ill with COVID-19, and the weighted average percentages, through calculating the sum of the possibilities of loss of work multiplied by the number of people surveyed, then dividing that by the sum of the people surveyed.

Based on the weighted percentage of people aged over 18 unable to work in the surveyed groups, we projected the relationship between an individual *i*’s age and possibility of being unable to work as:

(1) $$U_{i}=3.1889-0.0466\;\mathrm{age}\;i\geq 18$$

An individual *i*’s employment possibility at different ages was calculated as:

(2) $$E_{i}=1-0.01\times U_{i}\;i\geq 18$$

### 2.3. Survival Function

To obtain the survival function, estimates of mortality rates and other quantities were taken from the US life tables published by the National Vital Statistics System for the year 2018. We assumed that, in the absence of COVID-19, mortality conditions in 2020 would be similar or equivalent to those observed in 2018 and that the 2020 population age distribution would be equivalent to the 2018 population age distribution. The numbers of deaths from COVID-19 in the United States were obtained from the Centers for Disease Control and Prevention (CDC) [24]. The numbers used in the calculations were updated according to those released by the CDC on 1 January 2020. For deaths through to 28 August 2021; we used the projected deaths through to 31 December 2020 and produced the survival function, *S*_i_, for single-year age intervals from birth to 85 years and over for the index group ill with COVID-19. The survival function, *S_j_*, of the healthy population was calculated from the National Vital Statistics System for the year 2018 for single-year age intervals from birth to 85 years.

Incorporating the survival function with employment possibility, the weekly employment status of the index group at different ages was *S_i_E_i_* and that of the healthy group at different ages was *S_j_*. The ratio of the employment possibility of an individual *i* in the index group relative to that of an individual *j* in the healthy group was calculated as:

(3) $$Employment\;ratio=\frac{S_{i}E_{i}}{Sj}\;i,j\geq 18$$

**Figure 1:**
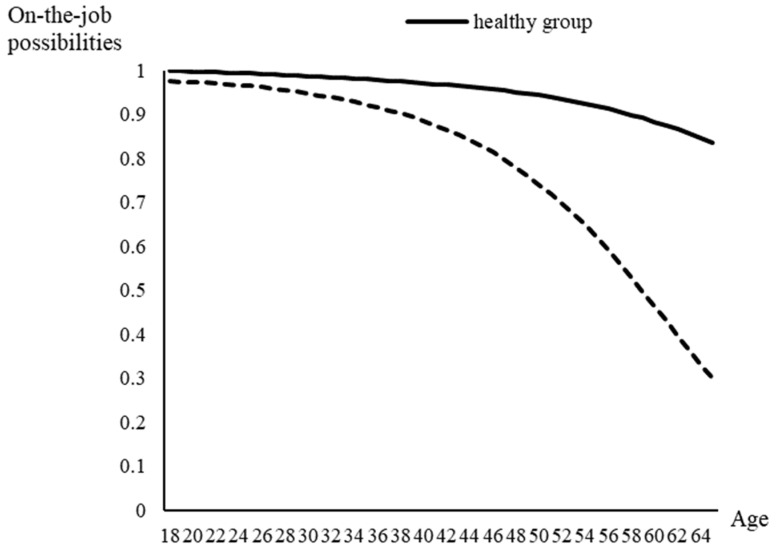
The weekly working probabilities for healthy adults aged 18–64 and those sick with COVID-19.

Based on the weighted percentage of people unable to work in each surveyed age group, there was a trend showing that the older the age group, the lower the on-the-job possibility. Figure 1 indicates that the differences in on-the-job possibilities between the index and the healthy group increased with age, especially for the group aged 45–64 years.

## 3. Results

### Employment Ratios and Productivity Loss Associated with COVID-19

The healthy population aged from 18 to 64 years formed a representative sample of the labor force. An individual *j*’s survival status, *S_j_*, was regarded as on-the-job possibility. The index group who were sick or quarantined, were in a much worse position to devote themselves to the labor market compared to when they were not infected. Individual *i* was employed only when she/he survived, presented by *S_i_*. An individual *i*’s on-the-job status in the index group was presented by *E_i_*. The weekly productivity loss associated with COVID-19 for the economy’s active workforce was composed of survival possibility, on-the-job status, and weekly wage, *W*:

(4) $$Productivity\;Loss=\sum_{j=18}^{j=64}S_{j}W-\sum_{i=18}^{i=64}S_{i}E_{i}W$$

**Figure 2:**
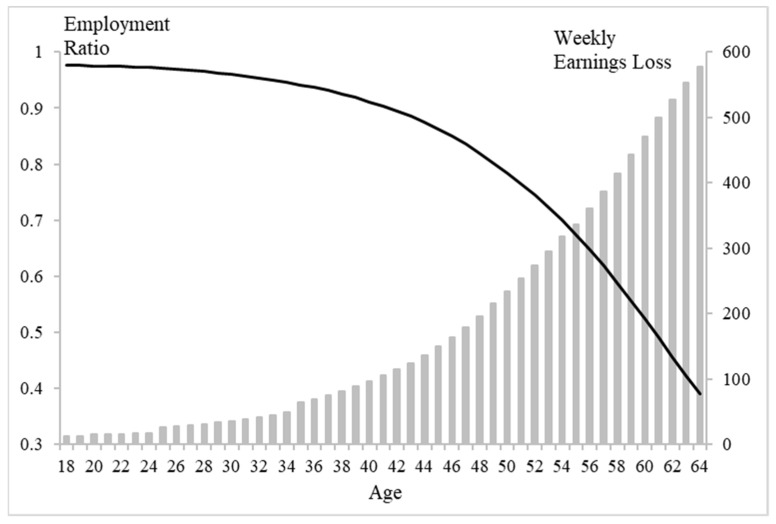
The weekly employment ratio of the group sick with COVID-19 relative to the healthy group and the weekly productivity loss for people aged from 18 to 64 years.

Figure 2 shows that the employment possibility of an individual *i* in the index group relative to that of an individual *j* in the healthy group, namely the employment ratio, decreased with age. The weekly productivity or loss in earnings due to self or a family member being sick from COVOID-19 increased with the individual’s age. For the group aged 45–64 years, the employment ratio of the index group relative to the healthy group decreased from 0.863 to 0.39 and the weekly productivity loss increased from 136.36 US dollars to 590.05 US dollars, as shown in Table 1 and Figure 2.

The analysis of the survey period reveals a lot about the sources of human capital and differences in earnings. When the working population’s health condition, presented by the survival function and employment status, exceeded those of the index population, their relative earnings increased.

## 4. Discussions and Policy Implications

Conventional economic evaluations on the effects of global crises, including pandemic disease, typically use variations in GDP or per capita GDP as indicators [22,25]. The GDP of an economy is generally influenced by business cycles and availability and/or the productivity of human capital. The outbreak of COVID 19 in the winter of 2019 raised the specter of a new, unknown and uncontrollable infectious disease that spread quickly and led to decreases in certain branches of economic activity [26,27]. At the same time, the unemployment rate in 2019 for the population of 18 years and over was around 4.5 per cent but increased sharply in 2020 to 9.18 per cent due to the pandemic in the US [28]. One of the major determinants of the elevated unemployment rate could be that the infected or quarantined groups were unable to work or might have been partially confined to working at home or in isolated places rather than in the company or factory. The shift in working locations as well as the monitoring of health status through telecommunication systems were important factors which might have enabled some of the index group to work for earnings [29].

With a given amount of physical capital and technology, an economy’s real GDP depends on the size of the labor force. Based on the US Bureau of Labor Statistics, the whole population was around 330 million, the population aged 18–64 years was around 210 million, the labor force participation rate was around 60 per cent, and the working force was around 126 million in 2020 [28]. We calculated the productivity loss for when an individual was unemployed due to self or a family member being sick from COVID-19. Based on the calculations from those aged 18 years and above in Table 1, the weekly mean productivity loss for the labor force was around 37.8 billion US dollars. When this loss was divided by the whole population, per capita productivity loss per week was around 115 US dollars. This translates into an annual per capita productivity loss of around 5956 US dollars, which would represent about 9 per cent of the per capita GDP, 65,055 US dollars, in 2020. We were concerned with the labor force and we did not estimate the productivity loss of other inputs, which may lead to a slight underestimation of the overall COVID-19 burden from an economic perspective.

### 4.1. Comparative Analysis of the Studied Phenomenon Compared with Other Countries

Recent studies have shown that on the basis of different assumptions of GDP loss, the lockdown caused by COVID-19 in the United Kingdom was associated with a loss of 91 billion US dollar in the best case and in the worst case with 729 billion US dollar [18,19]. The estimation corresponded to per capita GDP loss ranging from 1354 US dollars to 10,845 US dollars, or, 3 per cent to 27 per cent of the per capita GDP, which was 40,284 US dollars, in 2020 in the United Kingdom. Although these studies did not directly estimate the human capital loss and cannot be directly compared to our study, they represented the possible magnitudes of impact on GDP resulting from COVID-19 under different assumptions.

Based on the observed period between 30 January 2020 and 28 April 2020 in Italy, Nurchis et al. estimated that the total permanent productivity loss due to premature death was around 339 million US dollars, while the temporary productivity loss due to losing a job was around 113 million US dollars. In other words, the expected annual productivity loss was around 1808 million US dollars and approximately a 1% GDP loss. But this evaluation did not include other costs related to lockdown, quarantine of contacts, direct healthcare costs and so on [13], and could be considered as the lower bound of productivity loss. 

Facing the unprecedented and unpredictable disaster of the pandemic, health policies should be re-engineered [14,21,30]. Traditionally, the establishment of telehealth infrastructure could complement conventional in-person healthcare provisions [22,23,31,32,33]. As physically fit people would are able to work remotely or under safe physical social distance had they been supported by adequate telecommunication systems, a proactive provision of such infrastructure would possibly reduce human capital loss. Thus, in addition to vaccinations and contact tracing, future research on disaster preparedness may include providing telecommunication systems to vulnerable employees, such as those who are middle and old aged and/or comorbid with diabetes [24,34]. In fact, such efforts may have positive impacts on the population’s health and human capital productivities, even under the yearly threat of the flu [25,35].

### 4.2. Qualitative Analysis of Other Factors in the Application in Reality

The COVID-19 pandemic has had severe impacts on population health and the overall economy. The role of seasonality in the spread of this pandemic is imperative to public health interventions [36]. Uncertainty of the disease tends to increase during the winter months. On the other hand, interruptions to the functioning of the global supply chains have disrupted the access to raw materials [37]. Moreover, the increased health hazard has limited communication, trade and access to all kinds of goods and services along with market volatility and economic uncertainty. Recipients have distorted usual consumption patterns; and industries have created market anomalies. These elements of productivity have certainly affected economic development. According to OECD statistics, the global GDP decreased by 4.2% in 2020 and the recovery process will vary across different countries [38].

## 5. Conclusions

Health outcomes have been used for cost-effectiveness analysis, including productivity loss. Based on experiences of the United Kingdom, a three-month lockdown was associated with health benefits, more specifically, an additional 22,600 US dollars for each death avoided [19]. Our analysis is novel in that it estimates the burdens of COVID-19 in the US, underlining the importance of its impact on the productivity capacity of the labor force. The results of this study suggest that the working population’s health condition is one of the main determinants of productivity or earnings. Infected or quarantined groups would be confined to working in relatively isolated offices or places that allow for social distancing. Proactive health promotion at the workplace plus prompt reactive arrangements of work through telecommunication systems would probably reduce such losses. The infrastructure related to telehealth/e-health/telemedicine would be an important investment in times of the pandemic [39]. Notable effects of the pandemic on increased global health expenditure, including the influence of new variants on cases, hospitalization, and widespread vaccination, are expected [40]. How the pandemic will evolve and affect future health care spending deserves our attentions. This study could guide decision makers worldwide to timely implement cost-effective prevention and invest in infrastructure to tackle pandemics in the future.

### Limitations and Future Works

This study has the following limitations that must be acknowledged: First, we measured the population’s productivity loss based on human capital calculation but did not conduct longitudinal estimations because the official statistics of public health and macroeconomy were only published from 2020 to date. Using only the survey data at three time periods, our estimation would be relatively crude and would correspond to the period when vaccines and effective treatments were still unavailable. In other words, future studies are warranted to combine the transmission model with more frequent survey data for a more comprehensive understanding of the dynamic changes of such losses, especially when vaccines and/or effective treatments are available. Second, we regarded the survival function as a health indicator, which is composed of life expectancy and lifetime survival probability. The main goal of this research was not concerned with the clinical vulnerability and/or comorbidities of the sampled subjects nor the sex-based differences across age groups (since relevant data were not available) which RANDS could include in future surveys. Moreover, because the surveyed data by RANDS did not include a large sample size, neither did it contain any information on telecommunication and/or seasonality, one must be cautious when generalizing our results. Nevertheless, telehealth appointments constituted 8% of all medical appointments in 2019 in the U.S.A., which was found to increase by 683% between 2 March and 14 April 2020 during the COVID-19 pandemic [31]. We conjecture that such a quick jump on demand was associated with the COVID-19 pandemic to possibly mitigate the adverse impacts on health and the economy. We thus conclude that our study provides a quantitative characterization of the spill-over effects for the working population (stratified by age) and the burdens of a new pandemic infectious disease without vaccine nor effective treatment.

## Figures and Tables

**Table 1:** Weekly productivity loss and employment ratio of the group unable to work due to self or a family member with COVID-19 relative to the healthy group.

Age Group	9 June 2020–6 July 2020		3 August 2020–20 August 2020		17 May 2021–30 June 2021		Weighted Percentage of People Unable to Work
	Sample Size	Percentage	Sample Size	Percentage	Sample Size	Percentage	
18 years and above	6794	0.9	5974	1.1	5450	1.2	1.1
Weekly productivity loss (US dollars) ^1^	21.41–617.70		26.27–612.75		25.04–610.66		24.25–613.70
Employment ratio ^2^	0.979–0.0652		0.974–0.0653		0.975–0.065		0.976–0.0652
18–44 years	2606	1.5	2214	1.9	1975	1.9	1.75
Weekly productivity loss	10.81–135.13		13.16–137.03		14.03–136.91		13.52–136.36
Employment ratio	0.979–0.876		0.974–0.873		0.975–0.873		0.976–0.814
45–64 years	2386	0.5	2083	0.6	1735	0.8	0.62
Weekly productivity loss	148.32–589.51		152.04–594.93		150.57–587.71		136.36–590.05
Employment ratio	0.865–0.391		0.863–0.390		0.862–0.389		0.863–0.39
65 years and over	1802	0	1677	0.1	1740	0.4	0.17
Weekly productivity loss	544.98–627.56		538.74–624.15		530.52–610.05		530.09–619.25
Employment ratio	0.359–0.0652		0.358–0.0653		0.357–0.065		0.358–0.0652

Data sources: https://data.cdc.gov/NCHS/Loss-of-Work-Due-to-Illness-from-COVID-19/qgkx-mswu, accessed on 1 September 2021. ^1^ The calculations of weekly productivity loss are based on the weekly wages over the surveyed dates. ^2^ The employment ratio is the on-the-job possibility of the group sick with COVID-19 relative to that of the healthy group.

## Data Availability

The data underlying the results presented in the study are from third party web resources and are available from the URL: https://www.bls.gov/bls/news-release/wkyeng.htm#2020 (accessed on 1 September 2021). The statistics are open for the public.
